# Monuments to Jenner

**Published:** 1858-09

**Authors:** 


MONUMENTS TO JENNER.—It is a notable fact, that in London, where the charms of a pretty face are so much appreciated, especially among aristocratic circles, that even there Jenner's name has been but seldom mentioned since his decease, thirty-five years ago. It has not been so in some other lands, for, in the year 1821, a handsome monument to Dr. Jenner stood in the great square of Guatemala, a magnificent city in Central America. It is the capital of the State of that name, and has, or had at the above date, a population of 50,000 souls. The city stands about 8000 feet above the Pacific Ocean, on the western slope of the Andes, and contains an ancient and distinguished university. There the name of Jenner was honored and revered, even during his lifetime, as a benefactor to his species, and more worthy of a niche in our esteem than many of those who have been lauded as the conquerors and destroyers of mankind.—*Glasgow Herald.—Lancet*, June 5, 1858.

